# Challenges and Limitations of Anti-quorum Sensing Therapies

**DOI:** 10.3389/fmicb.2019.02473

**Published:** 2019-10-31

**Authors:** Paweł Krzyżek

**Affiliations:** Department of Microbiology, Wroclaw Medical University, Wrocław, Poland

**Keywords:** quorum sensing, quorum quenching, microbiota, pathogenicity, virulence, resistance

## Abstract

Quorum sensing (QS) is a mechanism allowing microorganisms to sense population density and synchronously control genes expression. It has been shown that QS supervises the activity of many processes important for microbial pathogenicity, e.g., sporulation, biofilm formation, and secretion of enzymes or membrane vesicles. This contributed to the concept of anti-QS therapy [also called quorum quenching (QQ)] and the opportunity of its application in fighting against various types of pathogens. In recent years, many published articles reported promising results indicating the possibility of reducing pathogenicity of tested microorganisms and their easier eradication when co-treated with antibiotics. The aim of the present article is to point to the opposite, negative side of the QQ therapy, with particular emphasis on three fundamental properties attributed to anti-QS substances: the selectivity, virulence reduction, and lack of resistance against QQ. This point of view may highlight new directions of research, which should be taken into account in the future before the widespread introduction of QQ therapies in the treatment of people.

## Introduction

Quorum sensing (QS) is a mechanism allowing microorganisms to sense population density, synchronously control genes expression after reaching a critical point (a quorum) and assess the efficiency of producing diffusible extracellular effectors. This process is associated with the synthesis of extracellular autoinductive substances with a function similar to hormones produced by higher organisms (Redfield, 2002; Hense et al., 2007; Bandara et al., 2012; Hawver et al., 2016). The first scientific report, indicating the presence of hormone-like compounds in bacteria, was an article by Tomasz (1965). In the next decade, attention was drawn to the relationship between the production of a specific group of metabolites (autoinducers) and luciferase-dependent bioluminescence in bacteria of the Vibrionaceae family (Nealson et al., 1970; Greenberg et al., 1979). A breakthrough in QS research was made by Eberhard et al. (1981), who for the first time identified the structure of signaling factors involved in the microbial communication, i.e., acyl-homoserine lactones (AHLs), now often named as autoinducer-1 (AI-1). In 1994, the presence of other substances controlling bioluminescence was shown (Bassler et al., 1994). They were called autoinducer-2 (AI-2), and their structure was identified at the beginning of the twenty-first century (Chen et al., 2002). In 1995, two publications were released, in which the relationship between oligopeptides synthesis [called autoinducing peptides (AIPs)] and inter-microbial communication in Gram-positive bacteria was noticed (Havarstein et al., 1995; Ji et al., 1995). AI-1, AIPs, and AI-2 are currently the most intensively investigated compounds related to the QS activity, and their presence has been demonstrated in many bacteria belonging to Gram-negative, Gram-positive, and various classes of microorganisms, respectively (Bandara et al., 2012). In the following years, the presence of other substances associated with the microbial communication was also documented, including autoinducer-3 (AI-3) (Sperandio et al., 2003), *Pseudomonas* quinolone signal (PQS) (Deziel et al., 2004) and diffusible signal factors (DSFs) (Tang et al., 1991). In order to broaden the knowledge on the functioning of QS systems at the molecular level, we refer to the Hawver et al. (2016) review paper.

Quorum sensing controls the activity of many mechanisms important for the microbial physiology, including production of biofilm (Parsek and Greenberg, 2005; Dickschat, 2010), exoenzymes (Pena et al., 2019), membrane vesicles (Kulp and Kuehn, 2010; Toyofuku, 2019), siderophores (Cornelis and Aendekerk, 2004), and secondary metabolites with antimicrobial activity (Barnard et al., 2007; Kareb and Aïder, 2019), as well as induction of sporulation (Schultz et al., 2009), swarming motility (Daniels et al., 2004), and competence for horizontal gene transfer (Blokesch, 2012; Shanker and Federle, 2017). Because many of these processes are associated with virulence, there is a belief that inhibition of QS activity [also called quorum quenching (QQ)] will reduce pathogenicity and contribute to easier eradication of microorganisms. Examples of *in vitro* and *in vivo* studies showing the effectiveness of QQ substances in reducing virulence mainly include an activity of lactonase, an enzyme breaking down the lactone ring of molecules involved in QS (Fan et al., 2017; Guendouze et al., 2017; Rehman and Leiknes, 2018; Utari et al., 2018; Mion et al., 2019) and azithromycin, a macrolide antibiotic with QQ properties (Nalca et al., 2006; van Delden et al., 2012; Zeng et al., 2016). Currently, *in silico* studies are also used to search for new, promising QS inhibitors to accelerate the effectivity and reduce the costs associated with the discovery of new compounds with such properties (Mellini et al., 2019). Promising features of QQ substances are reflected in the presence of many review papers describing the possibilities resulting from the use of this type of compounds (LaSarre and Federle, 2013; Chen et al., 2018; Defoirdt, 2018; Rémy et al., 2018; Fleitas Martínez et al., 2019).

The aim of this article is to point to the opposite, negative side of the QQ therapy, with particular emphasis on three fundamental properties attributed to anti-QS substances: the selectivity, virulence reduction, and lack of resistance against QQ.

## The First Objection – The Selectivity of Quorum Quenching Substances

**Figure 1:**
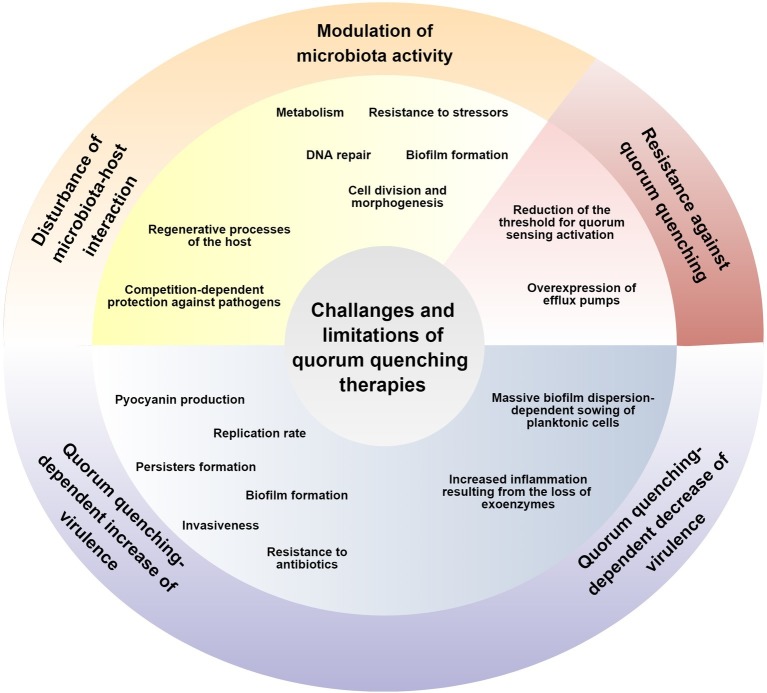
Challenges and limitations of anti-QS therapies are marked by yellow, blue, and red colors for the selectivity, reduction of microbial virulence, and lack of the possibility to develop resistance mechanisms, respectively.

Despite the key role of QS signals in the virulence of many pathogens, the involvement of these signaling factors in the physiological processes of microorganisms is rarely taken into account. For AI-2, participation in controlling gene expression related to metabolism (DeLisa et al., 2001; McNab et al., 2003; Shao et al., 2012; Mitra et al., 2016; Ha et al., 2018; Yadav et al., 2018), cell division, and morphogenesis (DeLisa et al., 2001; Shao et al., 2012; Yadav et al., 2018), and DNA repair (Yadav et al., 2018) has been demonstrated. Importantly, the presence of the AI-2 producing *luxS* system was also noticed in commensal bacteria inhabiting the human body, including *Bifidobacterium* (Sun et al., 2014) and *Lactobacillus* (Lebeer et al., 2007; Liu et al., 2017, 2018a), but also many other representatives, such as *Eubacterium*, *Roseburia*, or *Ruminococcus* (Lukáš et al., 2008). Signaling associated with AI-2 in these bacteria is associated with adaptation to environmental conditions, affecting biofilm formation (Rickard et al., 2006; Lebeer et al., 2007; Cuadra-Saenz et al., 2012; Sun et al., 2014; Liu et al., 2017, 2018a) and resistance to stressors during the passage through the digestive tract (Yeo et al., 2015; Liu et al., 2018a). Additionally, the *luxS* system is required for the synthesis of bacteriocins by *Escherichia coli* (Lu et al., 2017), *Streptococcus pneumoniae* (Miller et al., 2018), *Streptococcus mutans* (Merritt et al., 2005; Sztajer et al., 2008), and *Lactobacillus* (Jia et al., 2017; Li et al., 2019). It was observed that AI-2 produced by *Aggregatibacter actinomycetemcomitans* is a factor directly involved in the inhibition of both biofilm formation and transformation into the filamentous form by *Candida albicans* (Bachtiar et al., 2014). Similarly, the production of these signaling factors was crucial in the *Ruminococcus obeum*-dependent reduction of intestinal colonization by *Vibrio cholerae* (Hsiao et al., 2014) or *Bifidobacterium*-dependent protection against *Salmonella* infections (Christiaen et al., 2014b). Therefore, it should not be surprising that the disruption of AI-2-related signaling may have an indirect/direct effect on the ability of human microflora to adhere, form biofilms, and produce metabolites with antimicrobial activity and hence results in disturbance of microbiota homeostasis (Figure 1; Thompson et al., 2016). The use of enzymes degrading QS molecules, instead of chemicals inhibiting QS (5-fluorouracil or brominated furanones) seems to be a solution because the former show higher selectivity against targeted microorganisms (Chen et al., 2013; Guendouze et al., 2017; Liu et al., 2019).

Thompson et al. (2015), in pioneer studies determining the effect of AI-2 on intestinal microflora of antibiotic-treated mice, showed that a modified *E. coli* strain producing AI-2 promoted the expansion of the *Firmicutes* phylum and increased the *Firmicutes*/*Bacteroides* ratio in guts. The authors of the article suggested that the use of antibiotics may most likely contribute to the destruction of microorganisms belonging to *Firmicutes* and create an environment with a low AI-2 concentration (Thompson et al., 2015). Other authors in their report also pointed to the beneficial, buffering effect of AI-2 on the number of *Firmicutes* in stool samples treated with this signaling factor (Park et al., 2016). In another study, it has been shown that lactonase has the modulating effect on the composition of biofilm and planktonic soil microorganisms. The most significant changes were observed for *Stenotrophomonas* and *Pseudomonas* (increase), as well as *Clostridium* cluster XIVa (decrease) (Schwab et al., 2019). Although the experiment was conducted against soil bacteria, these observations indicate the possibility of quantitative changes in microorganisms exposed to factors limiting the QS activity. Particularly, worrying is the decrease in the amount of butyrate-producing *Clostridium* clade XIVa, constituting about 60% of the mucosa-associated microbiota within the gut (Van den Abbeele et al., 2013). In many scientific reports, a relationship between the decrease in the number of these bacteria and the development of pro-inflammatory diseases, including cystic fibrosis (Duytschaever et al., 2013), sclerosis (Miyake et al., 2015), irritable bowel disease (Sokol et al., 2006; Imhann et al., 2018), and an increase in the amount of *Enterococcus* (Livanos et al., 2018) and *Clostridium difficile* (Antharam et al., 2013) in intestines, has been observed. Anti-QS therapies could thus adversely affect the number of *Clostridium* cluster XIVa and contribute to the formation of pro-inflammatory/autoimmune diseases.

The impact of QS on the human health may be associated not only with the modulation of microbiota metabolic activity but also with the direct effect of signaling factors on the host. It was determined that AHLs are chemoattractants for neutrophils (Smith et al., 2002; Zimmermann et al., 2006; Karlsson et al., 2012). In addition, many *in vivo* and *in vitro* studies have demonstrated the direct involvement of AHLs in inducing pro-inflammatory (Smith et al., 2002; Li et al., 2009; Grabiner et al., 2014; Curutiu et al., 2018; Huang et al., 2018) and pro-apoptotic (Schwarzer et al., 2012, 2015; Losa et al., 2015) responses in eukaryotic cells. However, in studies using the skin wound healing model, it was observed that the AHLs-dependent pro-inflammatory activity of neutrophils is associated with the differentiation of fibroblasts into myofibroblasts and is crucial for tissue regeneration (Nakagami et al., 2011; Kanno et al., 2013, 2016). The ability of AHLs to stimulate protective, innate immune responses was also detected in mice treated with these compounds because exposure of mice to AHLs increased their survival during infection with *Aeromonas hydrophila* (Khajanchi et al., 2011). For AI-2, the potential to stimulate immune cells and increased secretion of pro-inflammatory cytokine IL-8 was also indicated (Zargar et al., 2015). On the other hand, the host can also affect the activity of the microflora. Ismail et al. (2016) observed that mammalian epithelial cells produce AI-2 mimics in response to the presence of bacteria. Because the synthesis of these molecules occurred during the destruction of tight junctions, the authors of the manuscript concluded that AI-2 mimics are probably involved in the stimulation of symbiotic microflora-dependent regenerative processes (Ismail et al., 2016). On this basis, it can be concluded that a risk of disrupting regeneration processes in the human body during anti-QS therapy may occur. This could develop by interfering with both the microflora QS activity and the AI-2 mimics-dependent host-microbiota signaling (Figure 1).

## The Second Objection – The Reduction of Virulence by Quorum Quenching Substances

One of the main assumptions of anti-QS therapy is the reduction of pathogens’ virulence by limiting the communication-dependent pathogenicity induction, reviewed in Defoirdt (2018) and Fleitas Martínez et al. (2019). This effect was observed in both *in vitro* and *in vivo* animal studies (Chu et al., 2013; Jang et al., 2013; Park et al., 2014; Ryu et al., 2016; Zhou et al., 2016; Kim et al., 2018; Torabi Delshad et al., 2018). There are, however, numerous scientific reports indicating that the dysfunction of genes responsible for the QS activity determines an increase of certain pathogenicity features. Among microorganisms in which deletion of *luxS* (Δ*luxS*) increased the aggregation or/and biofilm formation are representatives of Gram-negative bacteria: *Helicobacter pylori* (Cole et al., 2004; Anderson et al., 2015; Sweeney et al., 2018), *Vibrio cholerae* (Ali and Benitez, 2009), *Aggregatibacter actinomycetemcomitans* (Velusamy et al., 2017), *Actinobacillus pleuropneumoniae* (Li et al., 2008), and *Haemophilus parasuis* (Zhang et al., 2019), as well as Gram-positive: *Staphylococcus aureus* (Yu et al., 2012; Ma et al., 2017), *Staphylococcus epidermidis* (Xu et al., 2006; Xue et al., 2015), *Streptococcus mutans* (Huang et al., 2009; He et al., 2015), *Enterococcus faecalis* (He et al., 2016), and *Bacillus cereus* (Auger et al., 2006). In addition, Δ*luxS Streptococcus pyogenes* and *S. aureus* mutants showed an increase in survivability when incubating with macrophages (Siller et al., 2008; Zhao et al., 2010). The use of anti-QS therapy could therefore promote the development of isolates with an increased survival ability and are thus more difficult to eradicate (Figure 1).

Despite the aforementioned examples, there are many microorganisms for which the presence of the *luxS* gene and the production of AI-2 is crucial for the formation of biofilms (McNab et al., 2003; Jesudhasan et al., 2010; Jang et al., 2013; Li et al., 2015, 2017; Laganenka et al., 2016; Jani et al., 2017; Laganenka and Sourjik, 2018). The aim of QQ therapy, in this case, would be to maintain the microorganisms in a planktonic form, more sensitive to antibiotics or immune cells’ attack (Chow et al., 2014; Christiaen et al., 2014a; Ryu et al., 2016; Luo et al., 2017; Srinivasan et al., 2017; Mayer et al., 2018; Yu et al., 2018). In pioneer *in vivo* studies by Fleming and Rumbaugh (2018), for the first time, the effect of massive dispersion of bacteria using the mouse wound infection model was determined. It was noticed that the dispersion of biofilm resulted in the rapid release of planktonic bacteria and their sowing into the blood (Fleming and Rumbaugh, 2018). Such a scenario indicates a potential risk of developing bacteremia/sepsis as a consequence of the biofilm disruption during the QS inhibition (Figure 1).

*S. aureus* is one of the references, Gram-positive bacteria in studies determining the effect of QS on the physiology of bacterial cells. In addition to the aforementioned ability of AI-2 production, staphylococci has also another important QS system controlling their functioning, i.e., accessory gene regulator (Agr) (Le and Otto, 2015). In Δ*agr* mutants, a higher biofilm production (Vuong et al., 2004; He et al., 2019) and higher degree of persister forms development, a phenotype associated with changed metabolism and resistance to many antibiotics (Xu et al., 2017), were observed (Figure 1). For Δ*agr* mutants, an increased tendency to initiate a chronic, difficult to eradicate bacteremia was also indicated (Fowler et al., 2004; Paulander et al., 2012; Park et al., 2013; Kang et al., 2017). He et al. (2019) assessed the selective pressure associated with maintaining of the Agr activity in isolates from biofilm vs. non-biofilm staphylococcal infections. It has been observed that Δ*agr* mutants appear practically exclusively during the biofilm phase, so there is a high selective pressure to maintain the Agr system when staphylococci are present in the planktonic form. For this reason, according to the authors of the article, it seems that the use of QS inhibitors would be useful only in therapies of infections unrelated to biofilm formation (He et al., 2019). This condition, however, may be difficult to meet because it was estimated that nearly 80% of all chronic infections in the human body are associated with biofilms (Römling and Balsalobre, 2012).

*P. aeruginosa*, a representative of Gram-negative bacteria, is an alternative to *S. aureus* model microorganism in QS research. In this bacterium, two QS systems associated with the production of AHLs (Las and Rhl) are present (Lee and Zhang, 2015). In Δ*lasR* mutants (without an ability to detect signaling factors), a selective advantage related to the replication rate in the stationary phase (D’Argenio et al., 2007; Lujan et al., 2007), as well as the increased activity of β-lactamases (D’Argenio et al., 2007), as compared to the wild-type strain was noticed. Additionally, in a placebo-controlled trial, it has been shown that a QS-inhibiting antibiotic, azithromycin, increases the prevalence of *P. aeruginosa* strains with higher virulence after treatment with this drug (Köhler et al., 2010). In an *in vitro* study, it has been observed that Δ*lasR* mutants had an altered phenotype, i.e., a 4- to 12-fold higher production of pyocyanin and 1.5-fold greater motility, but had a significantly lower level of exoprotease and elastase secretion (Lujan et al., 2007). Another study found that the lower production of exoenzymes had its immunological consequences *in vivo*. In Δ*lasR P. aeruginosa* mutants, adapted to cystic fibrosis, the ability to induce more intensive host immune responses compared to the wild-type strain was observed. This mechanism was associated with an increased secretion of pro-inflammatory cytokines and neutrophils recruitment, as a result of the loss of exoenzymes-dependent cytokine degradation by *P. aeruginosa* Δ*lasR* mutants (LaFayette et al., 2015). This indicates the possibility of exacerbation of pro-inflammatory reactions at the site of ongoing infections after the use of QS inhibitors by limiting the number of microbial enzymes degrading mediators of inflammatory responses (Figure 1). On the other hand, it has been shown that oxidative stress, which could be associated with the inflammation-dependent generation of reactive oxygen species, contributes to the selection of *P. aeruginosa* cells having an active QS system (García-Contreras et al., 2015). Therefore, it seems that the final result may depend on the environmental conditions prevailing during the infection, the immune status of infected people, as well as the pathogenic potential of the specie/strain of the microorganism (Chugani et al., 2012; Feltner et al., 2016; Kostylev et al., 2019). A perspective article, pointing out the limitations and challenges facing the introduction of QQ therapies for treatment of *P. aeruginosa*, was written by García-Contreras (2016). The benefits of using QQ therapies against *P. aeruginosa* have been discussed in review papers by Chan et al. (2015) and Pérez-Pérez et al. (2017).

## The Third Objection – The Lack of Possibility to Develop Resistance Against Quorum Quenching Therapies

Another basic assumption of anti-QS therapies, apart from the selectivity and the reduction of microbial virulence, is the lack of the possibility for microorganisms to develop resistance mechanisms against this type of treatment. This postulate was based on the ability of QS inhibitors to disrupt systems controlling the pathogenicity of microorganisms and/or the lack of bactericidal activity of these compounds (Hentzer et al., 2003; Peters et al., 2003; Rasch et al., 2004; Rasmussen et al., 2005; Lönn-Stensrud et al., 2007; Swem et al., 2009; Ng et al., 2012; Park et al., 2014). The majority of research in which above-mentioned features were indicated came before 2012, the year in which for the first time the isolation of bacteria with reduced sensitivity to QS inhibitors was demonstrated. The first study, using computer modeling, determined the possibility of developing resistance to QQ by digital microorganisms by reducing the level of signaling factors needed to activate QS processes (Beckmann et al., 2012). In the same year, Maeda et al. (2012) in *in vitro* studies observed that *P. aeruginosa* could develop resistance to QS inhibitors (in this case, brominated furanones) by mutating genes encoding efflux pumps, proteins responsible for the removal of harmful substances from cells. These observations were confirmed in subsequent experiments conducted by the same research group (García-Contreras et al., 2013b). In 2018, it was shown that horizontal gene transfer in *P. aeruginosa* may be associated with the spread of integrative and conjugative elements responsible for resistance to both carbapenems and azithromycin-dependent inhibition of QS (Ding et al., 2018). Thus, contrary to the prevailing opinion, there is a possibility of developing resistance to QQ therapies (Figure 1). *In silico* modeling seems to be one of the potential solutions limiting the spread of resistance to QQ therapies (Wei et al., 2016). The topic of resistance against QQ therapies was discussed in details in review papers by Defoirdt et al. (2010), García-Contreras et al. (2013a), Kalia et al. (2014), and Liu et al. (2018b).

## Conclusion

Over 50 years have passed since the first observation indicating the ability of bacteria to produce hormone-like substances, referred to today as autoinducers. Since then, our knowledge about the universality and functions of QS in various groups of microorganisms has significantly expanded. Especially in the last two decades, thanks to the development of sophisticated genetic and microbiological techniques, scientists have been able to demonstrate the participation of QS in many key microbial processes, most of them related to pathogenicity. This dependence contributed to the concept of anti-QS therapy and the possibility of its application in fighting against various types of pathogens. This article is a voice in the discussion indicating the challenges and limitations facing such therapies. Its aim is not to lower the value of previously published papers but to point to potential new directions of research, which should be taken into account in the future before the widespread introduction of QQ therapies in the treatment of people.

## Data Availability Statement

No datasets were generated or analyzed for this study.

## Author Contributions

The author confirms being the sole contributor of this work and has approved it for publication.

### Conflict of Interest

The author declares that the research was conducted in the absence of any commercial or financial relationships that could be construed as a potential conflict of interest.
